# X-ray lines and self-interacting dark matter

**DOI:** 10.1140/epjc/s10052-015-3788-8

**Published:** 2015-11-30

**Authors:** Yann Mambrini, Takashi Toma

**Affiliations:** Laboratoire de Physique Théorique, Université de Paris-Sud 11, CNRS-UMR 8627, 91405 Orsay Cedex, France

## Abstract

We study the correlation between a monochromatic signal from annihilating dark matter and its self-interacting cross section. We apply our argument to a complex scalar dark sector, where the pseudo-scalar plays the role of a warm dark matter candidate while the scalar mediates its interaction with the Standard Model. We combine the recent observation of the cluster Abell 3827 for self-interacting dark matter and the constraints on the annihilation cross section for monochromatic X-ray lines. We also confront our model to a set of recent experimental analyses and find that such an extension can naturally produce a monochromatic keV signal corresponding to recent observations of Perseus or Andromeda, while in the meantime it predicts a self-interacting cross section of the order of $\sigma /m \simeq 0.1{-}1~\mathrm {cm^2/g}$, as recently claimed in the observation of the cluster Abell 3827. We also propose a way to distinguish such models by future direct detection techniques.

## Introduction

Dark matter is inferred to exist, through its gravitational interactions with visible matter, within and between galaxies [1–4]. Even if the PLANCK satellite [2, 3] confirmed that about 85 % of the total amount of the matter is dark, the community still lacks clear evidence of its nature through a direct or indirect signal. Indeed, the latest results of XENON100 [5], LUX [6], and FERMI observation of the galactic center [7, 8] or dwarf galaxies [9] impose very strong constraints on the mass of a weakly interacting massive particle, (if one excludes the 3$\sigma$ galactic center excess consistent with the range of dark matter identified in the FERMI-LAT data [10–12]), questioning the WIMP paradigm. Little is known about the mass and coupling of dark matter, and even the “WIMP miracle” is questionable [13] if one introduces a hidden mediator sector “*X*”, with its mass $m_X$ and coupling $g_X$ respecting $m_X/g_X \simeq m_\mathrm {wimp}/g_{EW}$ where $m_\mathrm {wimp}$ is the WIMP mass and $g_{EW}$ is the electroweak gauge coupling constant. Much lighter and warmer candidates are then allowed and can justify the lack of GeV signal in direct and indirect detection experiments, while explaining in the meantime recent claims at the keV scale [14].

A possible smoking gun signature of the interaction of dark matter in our galaxy or in larger structure would be the observation of a monochromatic signal (photon, neutrino or positron) generated by the annihilation or the decay of the candidate. In 2012, several studies claimed for the observation of a $135~\mathrm {GeV}$ monochromatic photon-line produced near the center of our Milky Way [15–18]. Phenomenological models describing the possibility of generating such a line then appeared in the literature [19–32]. More recently, the presence of a seemingly unexplained X-ray line observed by the XMM-Newton observatory in galaxies and galaxy clusters [33–35] increased the interest for annihilating [36, 37] or decaying [38–50] light dark matter scenarios. Excited dark matter [51–58] or axion-like candidates [59, 60] were also proposed as alternative interpretations.^1^

On the other hand, if the X-ray line excess discussed above is interpreted as a dark matter signal, the same excess should be observed from the other galaxies such as the Milky Way, M31, and dwarf spheroidal galaxies in addition to the Perseus and Centaurus clusters. However, such a signal has not yet been observed in Milky Way [62], M31 [63], stacked galaxy [64] and stacked dwarf galaxy [65] observations. For completeness, keeping open all possible interpretations of the 3.5 keV line signal, we will present the result of both analysis (signal or constraint) in every scenario we study in this work.

In parallel, recently the authors of [66] claimed that the observations of one (particularly well constrained) galaxy in the cluster Abell 3827 revealed a surprising $\simeq$1.62 kpc offset between its dark matter and stars. They affirm that such an offset is consistent with theoretical predictions from the models of self-interacting dark matter, implying a lower bound of the self-interacting cross section divided by the dark matter mass $\sigma /m \gtrsim 10^{-4}~\mathrm {cm^2/g}$. In the meantime, another group [67] with a different kinematical analysis for the very same galaxy obtained the value $\sigma /m\,\gtrsim 1.5~\mathrm {cm^2/g}$ in the case of contact interaction corresponding to the exchange of a massive mediator in opposition to long-range interaction which can arise for example from a massless mediator [68]. Entering into the debate of the exact value deduced from the observations is far beyond the scope of our work. However, one has to admit that any evidence for dark matter self-interaction would have strong implications for particle physics, as it would severely constrain or even rule out popular candidates such as supersymmetric neutralino/gravitino, axion, or any Higgs, *Z*, $Z'$ portal WIMP-like candidates. The main reason is that, within the sensitivity of present measurements, the observation of a self-interaction would imply the ratio $\sigma /m \simeq (10^{-5}$–$2) ~ \mathrm {cm^{2}\,g^{-1}} \simeq (0.05$–$9000)~\mathrm {GeV^{-3}}$, which is much larger than any typical WIMP values $\sigma _\mathrm {wimp}/m_\mathrm {wimp} \simeq 10^{-11}~\mathrm {GeV^{-3}}$.

In this work, we show that it is possible, in a minimal framework, to relate naturally the (smoking gun) monochromatic signal generated by the annihilation of a pseudo-scalar particle in its self-interaction process. As a consequence, any signal or constraint derived by the (non-)observation of self-annihilation (coming for instance from the “Bullet Cluster” (1E 0657-56) which is typically of the order of $\sigma /m \lesssim 1 ~\mathrm {cm^2 / g}$ [69, 70]) induces direct limits on the monochromatic signature. We begin our study by combining the constraints from different experimental analyses, before applying our results to the recent 3.5 keV line claims [33–35]. We show that the observation of such a signal implies naturally a relatively strong self-interacting process compatible with the limits on $\sigma /m$ obtained recently [66, 67]. We would like to insist that beyond the 3.5 keV signal consideration (one does not need to agree with the dark matter interpretation of the line or the self-interacting dark matter observations) the aim of our work is more general. We show the correlation which exists between an indirect detection signal and a self-interacting process once one builds an explicit microscopic model, with a dynamical symmetry breaking, which are not necessary present if one takes a pure effective approach.^2^

The paper is organized as follows. After a short description of the model under consideration in Sect. 2, we compute and analyze the self-interaction process combined with the monochromatic constraints and signal extracted from a set of different experimental collaborations in Sect. 3. Section 4 is devoted to the discussion and signatures in terms of indirect and direct detection prospects in more general cases. We draw our conclusions in Sect. 5, while an appendix contains alternative scenarios with fermionic dark matter.

## The framework

### Minimal model

In this section, we describe the model of a pseudo-scalar dark matter. The reader interested in alternative scenarios can find in the appendix the formulas in the case of fermionic dark matter. The model was originally built with success to interpret the recent monochromatic signal observed in different clusters of galaxies [36]. In this model, a scalar or pseudo-scalar particle is *by definition* a self-interacting particle. The Higgs boson, the unique observed spin 0 particle until now, is a self-interacting particle through its quartic coupling. Several other self-interacting candidates have been proposed in the literature, but usually these were spin 1/2 particles. However, in this case, it becomes necessary to invoke specific processes (like Sommerfeld enhancement, or strong interaction) to compensate for the dimensionality of the 4-fermion couplings. In the case of a scalar or pseudo-scalar dark matter $\phi$, the self-interaction term $\frac{\lambda }{4} |\phi |^4$ is always allowed by a global *U*(1) invariance and induces then necessarily self-interacting processes. Moreover, in the framework of spontaneous symmetry breaking, a strong correlation exists between the vacuum expectation value (vev) of $\phi$, its mass, and the quartic coupling $\lambda$, rendering the construction very predictive.

The general renormalizable potential for a scalar complex field $|\Phi |^2$ respecting a global *U*(1) symmetry is^3^

1 $$\begin{aligned} \mathcal {V}_{\Phi }=-\mu ^2|\Phi |^2+\frac{\lambda }{4}|\Phi |^4, \end{aligned}$$

where $\mu ^2$ is the bare mass of $\Phi$ and $\lambda$ is the quartic coupling of $\Phi$.

After a spontaneous breaking of the symmetry, it is straightforward to re-express the potential as a function of the fundamental components of $\Phi = v + \frac{s + i a}{\sqrt{2}}$ with $v= \langle \Phi \rangle = \sqrt{\frac{2}{\lambda }} \mu$. Absorbing the unphysical constants, we obtain

2 $$\begin{aligned} \mathcal {V}_{\Phi }= & {} \frac{m_s^2}{2} s^2 + \frac{\sqrt{\lambda }}{2 \sqrt{2}} m_s s^3 + \frac{\sqrt{\lambda }}{2 \sqrt{2}} m_s a^2 s \nonumber \\&+ \frac{\lambda }{16} s^4 + \frac{\lambda }{16} a^4 + \frac{\lambda }{8} a^2 s^2, \end{aligned}$$

with the scalar mass $m_s=\sqrt{2} \mu = \sqrt{\lambda } v$. It is important to notice that if our *U*(1) symmetry was exact (prior to developing a VEV), the pseudo-scalar dark matter mass $m_a$ would remain massless to all orders in perturbation theory. In the following, we will assume that the *U*(1) symmetry is broken by non-perturbative effects down to a discrete $Z_N$ symmetry. It is actually standard in string theory that all symmetries are gauged symmetries in the UV.^4^ Thus a non-zero dark matter mass $m_a$ being much lighter than $m_s$ is expected.

**Fig. 1:**
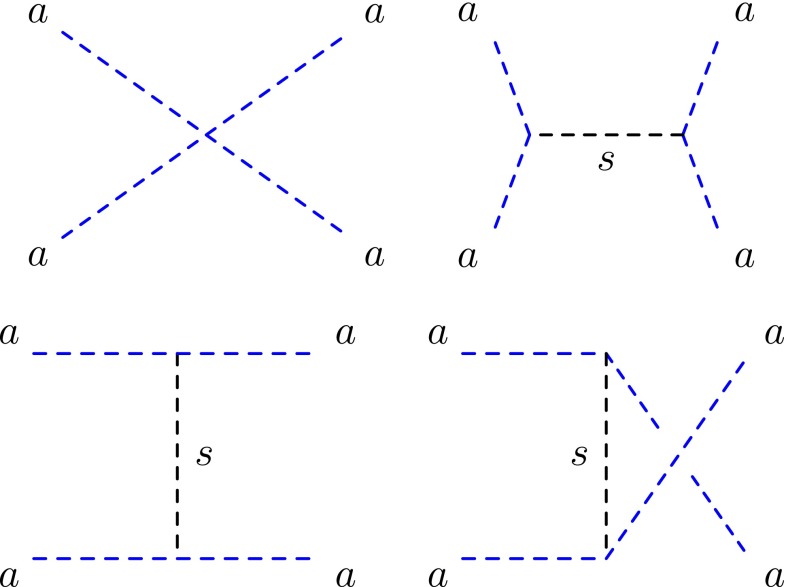
Feynman diagrams for dark matter self-interacting cross section

### The self-interaction process

In our model, we have four diagrams contributing to the self-interacting cross section as depicted in Fig. 1. Once the scalar part of $\Phi$ develops a VEV it becomes possible to re-express the total cross section as

3 $$\begin{aligned} \frac{\sigma _{aa}}{m_a}= & {} \frac{\lambda ^2 m_a}{32 \pi m_s^4 \left( 1-4 \frac{m_a^2}{m_s^2}\right) ^2} \nonumber \\\simeq & {} \frac{\lambda ^2 m_a}{32 \pi m_s^4},\quad (m_s \gg m_a). \end{aligned}$$

It is interesting to note that the cross section is of the form $$\sigma _{aa} \propto m_a^2/m_s^4$$ and then null for $$m_a=0$$, whereas if one takes into account only the quartic vertex *aaaa*, it should naively be proportional to $$1/m_a^2$$ and could potentially diverge. The mechanism canceling the divergences is in fact similar to the Higgs contribution occurring in the *WW* scattering in the Standard Model. This can easily be understood as $$m_a$$ can be considered as the pseudo-Goldstone boson generated by the breaking of the global *U*(1) symmetry. This fundamental feature *would not* have been observed in the framework of an effective approach if one introduces a dimensional coupling of the form $$\tilde{\mu }s aa $$, $$\tilde{\mu }$$ being a free mass parameter. It is thus the dynamical structure of the construction which defines precisely its self-coupling constants. Another interesting point is that, for a MeV scale mediator *s*, one does not need to invoke very large values of $$\lambda $$ to obtain a self-interacting cross section compatible with recent analysis. For instance, in the case of $$m_a = 3 $$ keV and $$m_s=1$$ MeV, one obtains $$\sigma _{aa}/m_a \simeq 7 \lambda ^2 ~\mathrm {cm^2 /g}$$, which is of the order of the measured limit ($$\sigma /m\lesssim 1~\mathrm {cm^2/g}$$) for a reasonable value of $$\lambda \simeq 1$$, much below the perturbativity limit, without invoking velocity enhancement.

### Monochromatic photon

Concerning the coupling to the photons, we consider the coupling which can be written as

4 $$\begin{aligned} \mathcal {L}_{s\gamma \gamma }=\frac{s}{\Lambda }F_{\mu \nu }F^{\mu \nu }, \end{aligned}$$

with $F_{\mu \nu } = \partial _\mu A_\nu - \partial _\nu A_\mu$ being the electromagnetic field strength. The scale $\Lambda$ can be interpreted in a UV completion since it can be determined by a set of new heavy charged particles running in triangular loops. The mass scale of new charged particles is assumed to be heavier than 300 GeV to respect the LEP constraint, depending on the number of charged fermions. Several experiments restrict $\Lambda$ from the Horizontal Branch (HB) stars processes [74–76] to the LEP [77] or beam dump experiment constraints [78, 79]. We will review them in detail in the next section, but roughly speaking, the coupling of a scalar to photons is extremely suppressed ($\Lambda \gtrsim 10^{10}~\mathrm {GeV}$) for $m_s \lesssim 300$ keV, largely due to the HB limits. For $m_s \gtrsim 300$ keV, a window opens, allowing values of $\Lambda$ as low as 10 GeV. In a UV complete model, such low values of $\Lambda$ can be understood if the number of fermions running in the loop is relatively important (of the order of 10).

**Fig. 2:**
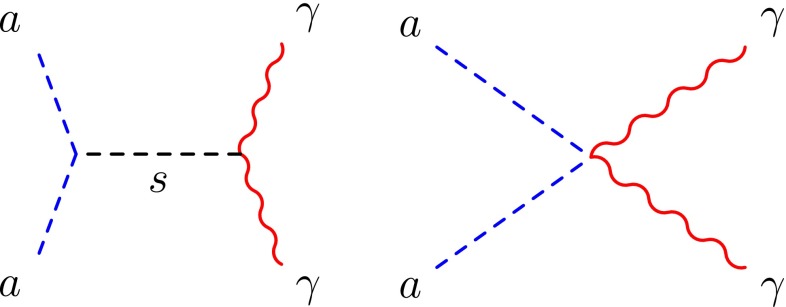
Feynman diagrams for dark matter annihilation into two photons. The second diagram can be generated by higher dimensional operators (see the text for details)

The presence of $s A_\mu A_\nu$ coupling generates naturally the production of monochromatic photons from the s-channel annihilation of the dark matter candidate *a* as depicted on the left of Fig. 2. The annihilation cross section for $aa\rightarrow \gamma \gamma$ is given by [36]

5 $$\begin{aligned} \sigma {v}_{\gamma \gamma }=\frac{\lambda m_a^2m_s^2}{\pi \Lambda ^2(m_s^2-4m_a^2)^2}. \end{aligned}$$

For $m_a\ll m_s$, the above cross sections, Eqs. (3) and (5), can be simplified to

6 $$\begin{aligned} \frac{\sigma _{aa}}{m_a}\approx \frac{\lambda ^2 m_a}{32\pi m_s^4},\quad \sigma {v}_{\gamma \gamma }\approx \frac{\lambda m_a^2}{\pi \Lambda ^2m_s^2}. \end{aligned}$$

By eliminating $\lambda$ in both expressions, it becomes possible for each energy $E_\gamma$ being equivalent to the dark matter mass $m_a$ since dark matter is almost at rest, to express $\sigma v_{\gamma \gamma } (E_\gamma )$*uniquely* as a function of $\Lambda$ and $\sigma _{aa}/m_a$,

7 $$\begin{aligned} \sigma v_{\gamma \gamma }= & {} \frac{4 \sqrt{2} E_\gamma ^{3/2}}{\Lambda ^2 \sqrt{\pi }} \sqrt{\frac{\sigma _{aa}}{m_a}}\nonumber \\\simeq & {} 1.3 \times 10^{-33} \left( \frac{100~\mathrm {TeV}}{\Lambda } \right) ^2 \left( \frac{E_\gamma }{3~\mathrm {keV}}\right) ^{3/2}\nonumber \\&\times \sqrt{\frac{\sigma _{aa}/m_a}{1~\mathrm {cm^2 /g}}} ~~ \mathrm {cm^3/s} . \end{aligned}$$

This is one of the main results of our work. It is indeed surprising that, asking for a reasonable value for the self-interacting cross section of the order of $1~\mathrm {cm^2/g}$, one obtains naturally the annihilation cross section of the order of $10^{-33}~\mathrm {cm^3\,s^{-1}}$ for a monochromatic keV signal, which corresponds exactly to the magnitude of the signals observed by XMM Newton [33–35] in the Perseus cluster.^5^ On the other hand, strong limits obtained from the non-observation of a monochromatic line by observatories such as HEAO-1 INTEGRAL, COMPTEL, EGRET, and FERMI restrict severely the lower bound on the scale $\Lambda$ in the rest of the parameter space, as we will analyze in the following section.

### A remark on higher-dimensional operator analysis

Building a complete ultraviolet model is far beyond the scope of this work, but we can give some hints for further developments. Indeed, even if the Lagrangian Eq. (4) breaks *explicitly* the *U*(1) symmetry, we can have a look at higher dimensional operators which can generate such a term after the breaking of the *U*(1) symmetry. The simplest dimension 6 operator can be written as

8 $$\begin{aligned} \mathcal {L}_{\Phi \gamma \gamma }=\frac{|\Phi |^2}{\tilde{\Lambda }^2}F_{\mu \nu }F^{\mu \nu }, \end{aligned}$$

with $\tilde{\Lambda }$ being a different cut-off scale from $\Lambda$ introduced in Eq. (4). After the symmetry breaking, one obtains the interaction terms

9 $$\begin{aligned} \mathcal {L}_{\Phi \gamma \gamma }\supset \left( \sqrt{\frac{2}{\lambda }}\frac{m_ss}{\tilde{\Lambda }^2}+\frac{a^2}{2\tilde{\Lambda }^2}\right) F_{\mu \nu }F^{\mu \nu }. \end{aligned}$$

One can then deduce from Eq. (9) the relation between $\Lambda$ and $\tilde{\Lambda }$: $\Lambda =\sqrt{\frac{\lambda }{2}}\frac{\tilde{\Lambda }^2}{m_s}$. The effective model built from the Lagrangian generates the second term in Eq. (9). This contact interaction contributes also to the annihilation cross section $aa\rightarrow \gamma \gamma$. Including this new contribution, the total cross section is then given by

10 $$\begin{aligned} \sigma {v}_{\gamma \gamma }=\frac{32m_a^6}{\pi \tilde{\Lambda }^4(4m_a^2-m_s^2)^2}. \end{aligned}$$

However, in the rest of our work we will continue to consider the dimension-5 coupling approach $\frac{s}{\Lambda } F^{\mu \nu } F_{\mu \nu }$ because a complete dimension-6 operator analysis would require a much more careful study of all the possible operators involved in the processes.

## The measurements

### Self-interacting dark matter

The status of the (non-)observation of self-interacting dark matter has become somewhat quite confusing recently, due to the release of (seemingly) contradictory results. Indeed, some authors of Ref. [66] using the new Hubble Space Telescope imaging, claimed to have observed that the dark matter halo of at least one of the central galaxies belonging to the cluster Abell 3827 is spatially offset from its stars. The offset, of the order of 1.62 kpc, could be interpreted as evidence of self-interacting dark matter with a ratio of cross section over mass of $\sigma /m \simeq 1.7 \times 10^{-4} ~\mathrm {cm^2/g}$.^6^ In the meantime, using a different kinematical approach from [66], the authors of [67] obtained a value of $\sigma /m \simeq (1.5$–$3)~\mathrm {cm^2/g}$, resulting in tension with the upper bounds set by other astrophysical objects such as the “Bullet Cluster” (1E 0657-56), which are typically of the order of $\sigma /m \lesssim 1 ~\mathrm {cm^2/g}$ [66, 69, 70, 80–82]. The main difference between the two analyses came from some approximations concerning the evolution time and from the gravitational back-reaction of the halo on its stars during the separation process due the drag forces. The authors of Ref. [67] have already addressed this issue some time ago in [83]. They clearly distinguished the contact interaction or *rare* interaction (our case) from the long-range force (involving Sommerfeld enhancement) or *frequent* self-interactions through the position of the peak of the dark matter distribution compared to the position of the stars/galaxies after the interaction.

In this work, we decided to take the two values proposed by the two groups as benchmark points, to show the correlation between an indirect signal (monochromatic photon in our case) and the self-interaction, once one has built an explicit microscopic model. Some recent phenomenological constructions explaining these observations can be found in [84] for a model with a gauged $Z_3$ discrete symmetry; a very nice interpretation in the framework of a Higgs portal (freeze-in mechanism) in [85, 86] whereas other authors introduced a dark photon sector [87, 88] or a strong interacting sector [89].

### Other experimental constraints

When a light (pseudo-)scalar interacts with photons, the helium burning period of HB stars is shortened due to non-standard energy loss since the light (pseudo-)scalar is produced in the stellar interior by photons within thermal distribution [74, 75]. This effect gives a strong constraint on the coupling between the light (pseudo-)scalar and photons for the (pseudo-)scalar mass lighter than $300~\mathrm {keV}$. The detailed analysis has been done in Ref. [76] and we used their result in our study.^7^

The interaction between the (pseudo-)scalar and photons is also constrained by the mono-photon search at leptonic collider experiments. Its signature is $e^+e^-\rightarrow \gamma + \mathrm{missing\,energy}$. The collider bound has been compiled in Ref. [74], taking into account the anomalous single photon (ASP) experiment [90]. In addition, the improved Large Electron-Positron Collider (LEP) limits based on the data of ALEPH, OPAL, L3, and DELPHI have been published in Ref. [77].

### Relic abundance

The computation of the relic abundance of dark matter in our framework has already been studied in detail in [36]. We will not repeat the analysis in this work, but we recall its main point. Adding interactions to the neutrino sector through *s* as a mediator can fulfill perfectly the relic abundance of dark matter measured by PLANCK while in the meantime generating naturally a massive neutrino sector respecting the recent cosmological bounds on neutrino masses ($m_\nu \lesssim 1$ eV; see [91] for a review on the subject). The presence of a dark bath between the neutrino and dark matter allows it in the parameter range of our work. However, keeping in mind this elegant possibility, our aim is to study the properties of self-interacting dark matter at the present time and the correlation between different observations in present large scale structures, independently on hypotheses concerning the thermal history generating the correct amount of dark matter abundance.

### The 3.5 keV line signal

Recent claims for a detection of X-ray line observed in galaxies and galaxy clusters like Perseus, by the XMM-Newton observatory [33–35] increased the interest in light dark matter scenarios. Keeping in mind that the status is still in debate (see the thermal atomic transition interpretation in [61] for instance), it is nevertheless interesting to apply our analysis in this concrete example to check if such a signal can be compatible with the limits derived from the recent self-interaction measurements.

The flux generated by the annihilation of dark matter in the Perseus cluster for instance can be computed from the luminosity of the cluster *L* [36]

11 $$\begin{aligned} L= & {} \int _{0}^{R_{Pe}} 4 \pi r^2 n^2_\mathrm {DM}(r) \langle \sigma v \rangle _{\gamma \gamma }\mathrm{d}r\nonumber \\= & {} \int _{0}^{R_{Pe}} 4 \pi r^2 \left( \frac{\rho _\mathrm {DM}(r)}{m_a} \right) ^2 \langle \sigma v \rangle _{\gamma \gamma }\mathrm{d}r, \end{aligned}$$

with the Perseus radius $R_{Pe}$, the number density of dark matter $n_\mathrm {DM}(r)$, the dark matter profile $\rho _\mathrm {DM}(r)$ and the thermally averaged cross section $\langle \sigma {v}\rangle _{\gamma \gamma }$. At a first approximation, one can consider a mean density of dark matter in the cluster as in Ref. [92]. The Perseus observation involved the mass of $M_{Pe}= 1.49 \times 10^{14} M_{\odot }$ in the region of $R_{Pe}=0.25$ Mpc at the distance of $D_{Pe}=78$ Mpc from the solar system. One can then estimate

12 $$\begin{aligned} n_\mathrm {DM}\simeq & {} \frac{1.49 \times 10^{14}M_{\odot } }{m_a}\!\bigg /\frac{4 \pi R_{Pe}^3}{3}\nonumber \\= & {} 1.9\times 10^{-37}~\mathrm {GeV^3}~~~(m_a=3.5~\mathrm {keV})\nonumber \\= & {} 2.5 \times 10^4~\mathrm {cm^{-3}}.\quad \end{aligned}$$

Combining Eqs. (11) and (12), one can then compute the luminosity in the Perseus cluster in the “mean” approximation $\langle L\rangle$,

13 $$\begin{aligned} \langle L\rangle\simeq & {} 1.2 \times 10^{55} \left( \frac{3.5~\mathrm {keV}}{m_a} \right) ^2\nonumber \\&\times \left( \frac{\langle \sigma v \rangle _{\gamma \gamma }}{10^{-26} \mathrm {cm^3\,s^{-1}}} \right) ~\mathrm {photon/s} . \end{aligned}$$

This estimation would be reasonable since dark matter in our model does not have a cusp profile such as NFW or Einasto, but a cored profile due to the large self-interacting cross section of dark matter. One can then deduce the flux $\phi _{\gamma \gamma } = L/(4 \pi D_{Pe}^2)$ that one should observe on earth,

14 $$\begin{aligned} \phi _{\gamma \gamma }= & {} 1.6 \times 10^{-5} \left( \frac{3.5 ~ \mathrm {keV}}{m_a} \right) ^2\nonumber \\&\times \left( \frac{\langle \sigma v \rangle _{\gamma \gamma }}{10^{-32} \mathrm {cm^3\,s^{-1}}} \right) ~\mathrm {cm^{-2}\, s^{-1}}. \end{aligned}$$

According to the authors of Refs. [33–35], one can identify the monochromatic signal arising from Andromeda galaxy (M31) or Perseus cluster with the flux $\phi _{\gamma \gamma }=5.2_{-2.13}^{+3.70} \times 10^{-5}~\mathrm {cm^{-2}\,s^{-1}}$ at 3.56 keV including the cluster core.^8^ We will parametrize our uncertainty from the dark matter distribution in the source by the classical “astrophysical” parameter $J_\mathrm{astro} \ge 1$ with $J_\mathrm{astro} = L/\langle L\rangle$, *L* being the effective luminosity for a steeper profile than the mean one we considered above.

Finally, extending the analysis by taking into account also other observations like M31, we will impose in our analysis a conservative annihilation cross section which is required to reproduce the X-ray line estimated as

15 $$\begin{aligned} \langle \sigma v \rangle _{\gamma \gamma } \simeq \frac{1}{J_\mathrm{astro}}(2 \times 10^{-33} {-} 8.5 \times 10^{-33}) \ \mathrm {cm^3\,s^{-1}}. \end{aligned}$$

## The results

### Combining the line and self-interaction

**Fig. 3:**
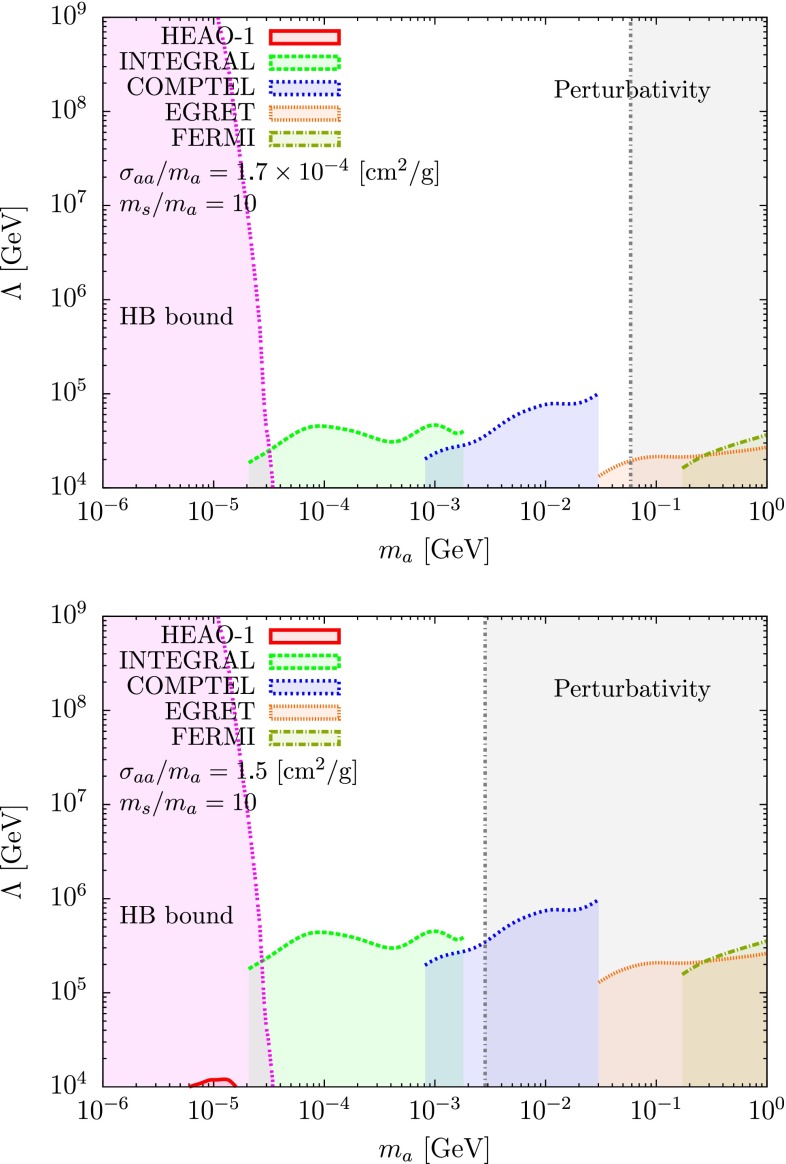
Limits on $\Lambda$ obtained from different observatories and satellites with the mass ratio fixed to $m_s/m_a=10$ where the white region is allowed and the colored region is excluded. The lower bounds of $\Lambda$ have been obtained from the data of satellites HEAO-1 (*red*), INTEGRAL (*green*), COMPTEL (*blue*), EGRET (*brown*), and FERMI (*dark-yellow*). The HB bound (*violet*) and perturbativity bound (*gray*) for $\lambda$ are also shown

**Fig. 4:**
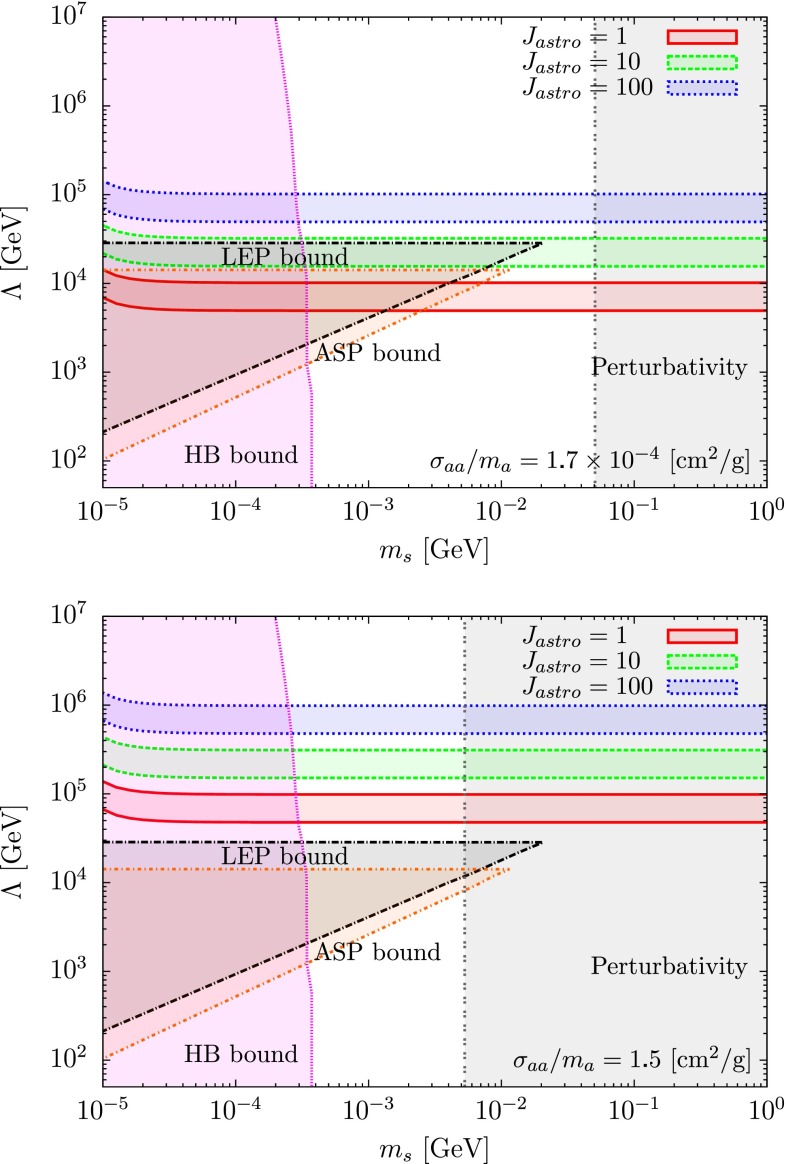
Parameter space ($m_s$, $\Lambda$) respecting at the same time the 3.5 keV line signal observed by XMM Newton [33–35] and two claimed values of self-interacting dark matter: $\sigma _{aa}/m_a=1.7\times 10^{-4}~\mathrm {cm^2/g}$ [66] (*above*), $\sigma _{aa}/m_a=1.5~\mathrm {cm^2/g}$ [67] (*below*). The factor $J_\mathrm{astro}$ corresponds to the astrophysical parameter. The values of $J_\mathrm{astro}=1~( red ),10~( green ),100~( blue )$ are taken ($J_\mathrm{astro}=1$ in the case of an isothermal profile). We also represented in the plot the actual limits from the HB star (*violet*), LEP (*black*), ASP (*brown*) and the perturbativity (*gray*)

We show in Figs. 3 and 4 the combined analysis, including the HB stars, ASP and LEP constraints [76, 77] for two different values of the self-interacting cross section, $\sigma _{aa}/m_a=1.7 \times 10^{-4}$ and $1.5 ~\mathrm {cm^2/g}$ corresponding to the values derived in [66, 67], respectively. In Fig. 3 the analysis is made by taking into account the current limits from different observations, whereas in Fig. 4 we fixed the annihilation cross section to fit the 3.5 keV line observation by XMM-Newton, Eq. (15).

We would like to insist that our aim is not to affirm that these two observations are the signatures of dark matter, but that combining these two physical measurements one can deduce a very strong constraint and/or information on $\Lambda$, especially if one uses the current limits from different experiments looking at the sky from the keV to the MeV energy range. To illustrate our purpose, we can extract lower bounds for the scale of the Beyond the Standard Model (BSM) $\Lambda$ from the observations of the satellites HEAO-1, INTEGRAL, COMPTEL, EGRET, and FERMI [93]. For our analysis, we required that the photon flux coming from the dark matter annihilation does not exceed the observed central value plus twice the error bar where the NFW dark matter profile is assumed [94]. This is depicted in Fig. 3 where we plot the limit we obtained on $\Lambda$ for $\sigma _{aa}/m_a = 1.7 \times 10^{-4} ~ \mathrm {cm^2/g}$ and $\sigma _{aa}/m_a = 1.5~\mathrm {cm^2/g}$ assuming the mass ratio $m_s/m_a=10$. We notice that the limits are quite stronger than the ones obtained by LEP, especially for a large self-interaction cross section.

As one can see from Fig. 4, it is interesting to note that there exists a band of parameter space, for $m_s \simeq$1–10 MeV and $\Lambda \simeq 10{-}1000$ TeV where one can explain the observed 3.5 keV line from the Perseus cluster for a self-interaction cross section of the order of magnitude of the one claimed to have been recently observed and still being largely compatible with accelerator searches.

### Non-detection of X-ray line

**Fig. 5:**
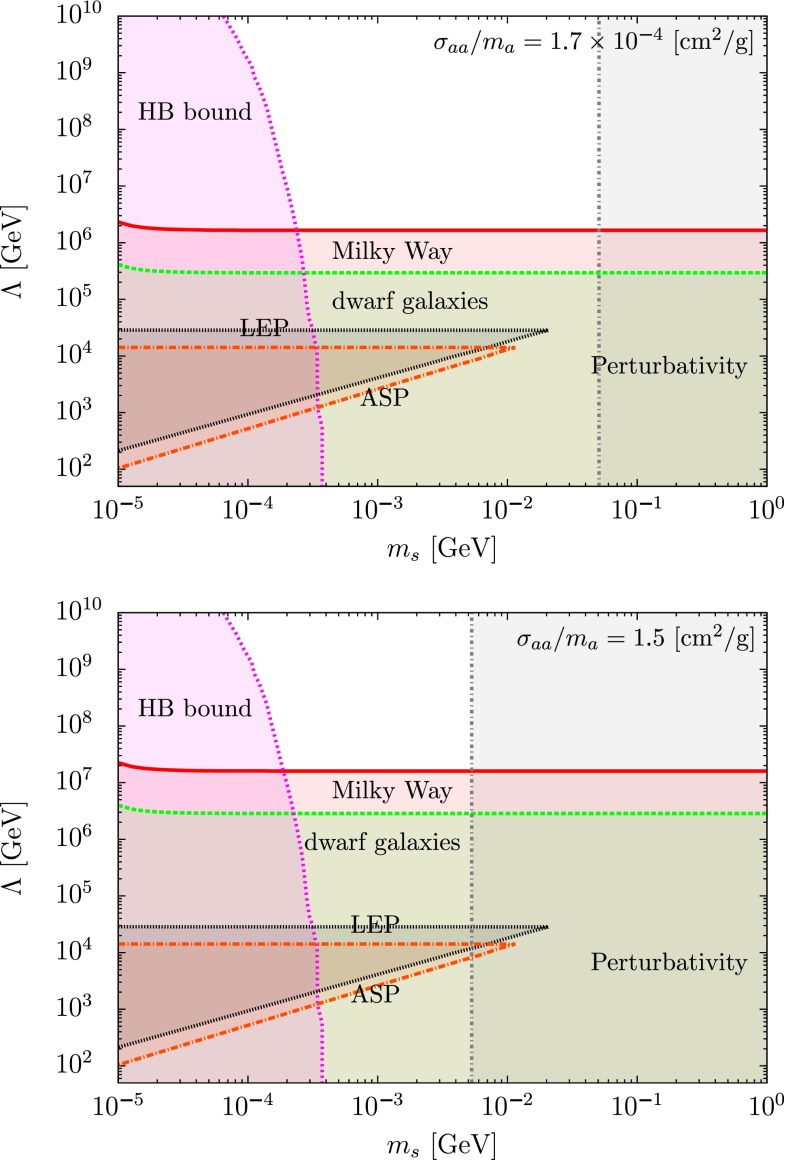
Same as Fig. 4 if one considers the non-observation of the 3.5 keV line in the Milky Way or dwarf galaxies. The region below the *red-full line* is excluded by the analyses of the Milky Way in [62], whereas the zone below the *green-dashed line* is excluded by the non-observation of the line in stacked dwarf galaxies [65]

If the $3.5~\mathrm {keV}$ X-ray line excess discussed above is interpreted as a dark matter signal, a same excess should be observed from the other galaxies such as the Milky Way, M31 and dwarf spheroidal galaxies in addition to the Perseus and Centaurus clusters. However, such an excess has not been observed for the Milky Way [62], M31 [63], stacked galaxies [64], and stacked dwarf galaxies [65]. For completeness, keeping open all possible interpretations of the 3.5 keV line signal, we present the result of these analyses in Fig. 5.

This inconsistency can be managed in some models. The first example is the scenario of decaying dark matter into an axion-like particle [95]. In this model, dark matter decays into an axion-like particle with the energy $3.5~\mathrm {keV}$. The axion-like particle produced in the process can be converted into photon via the astrophysical magnetic field around the galaxy clusters. Since the X-ray flux from dark matter depends on the strength of the magnetic field of each galaxy, the non-detection of the X-ray excess in some galaxies can be consistent.

Another type of interpretation concerns the possibility of an exciting dark matter  [96]. In this scenario, dark matter $\chi$ with the mass of the order of 10 GeV possesses an excited state $\chi ^*$. The excited state can be produced by up-scattering process $\chi \chi \rightarrow \chi ^*\chi ^*$ in the center of the cluster and converting the kinetic energy of dark matter. Then the excited state decays into the ground state and photon $\chi ^*\rightarrow \chi \gamma$. One can reproduce the X-ray line excess with the mass difference of $3.5~\mathrm {keV}$. Moreover, since the up-scattering process can occur in more massive and hotter environments such as clusters, non-detection of the X-ray line excess in smaller galaxies would be reasonable.

**Fig. 6:**
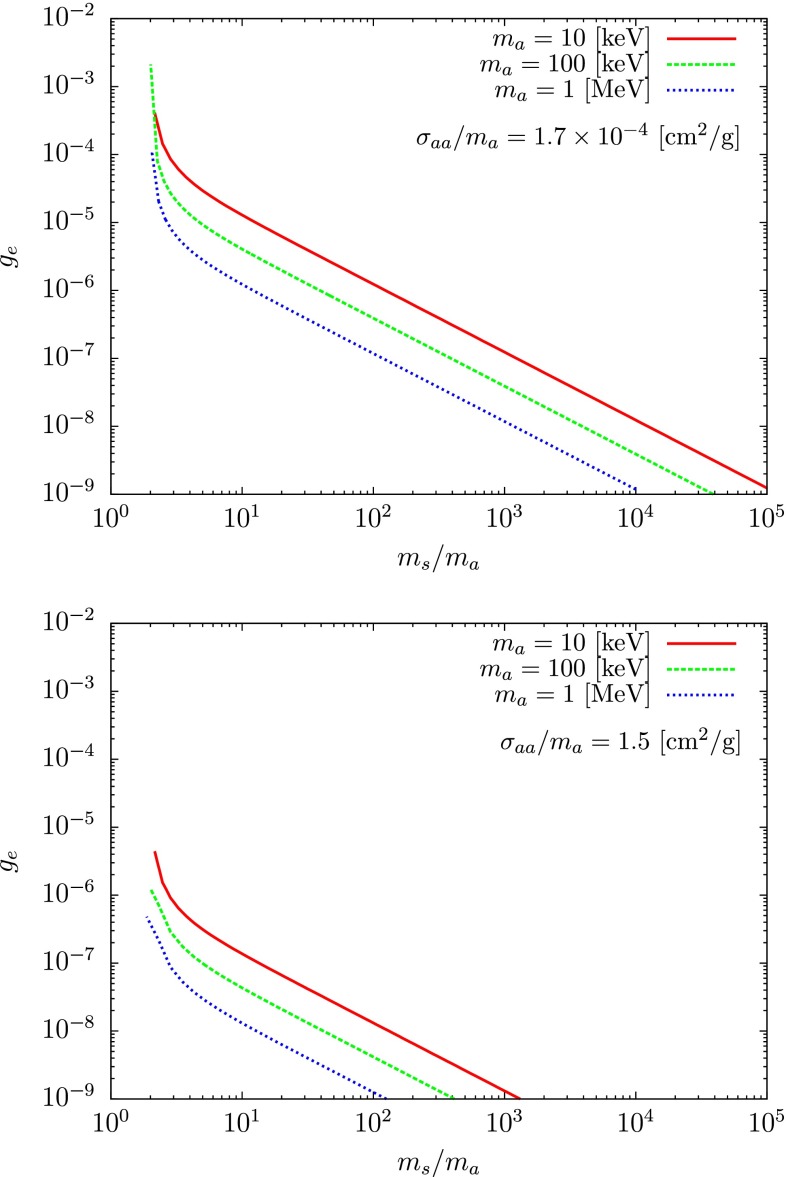
Limits on the electronic coupling to the scalar *s* as a function of the ratio $m_s/m_a$ for different values of $m_a$ and a ratio $\sigma _{aa}/m_a=1.7 \times 10^{-4}~\mathrm {cm^2/g}$ (*above*) and $\sigma _{aa}/m_a=1.5~\mathrm {cm^2/g}$ (*below*). The *curves* depict the sensitivity reach of the proposed superconducting detectors [97], for a detector sensitivity to recoil energies between 1 meV and 1 eV with a kg year of exposure

### Direct detection prospects

Such a light keV–MeV dark matter particle is clearly out of the reach of any present direct detection technology. However, recently, the authors of Ref. [97] proposed a new class of superconducting detectors which are sensitive to $\mathcal {O}$(meV) electron recoils from dark matter–electron scattering. Such devices could detect dark matter as light as 10 keV which is exactly the mass range of interest for our model. The idea is to observe the dark matter scattering off free electrons in a superconducting metal. Indeed, in a superconductor, the free electrons are bound into Cooper pairs, which typically have a meV scale (or less) binding energy, which is the typical energy transported by a $\mathcal {O}(10)$ keV dark matter with a local velocity $\simeq 300~\mathrm {km/s}$. Assuming that $g_e$ is the coupling of the electron to the scalar mediator *s*, one can straightforwardly compute the scattering cross section with an electron $\sigma ^e_{\mathrm {DD}}$:

16 $$\begin{aligned} \sigma ^e_{\mathrm {DD}}= \frac{\lambda ^2g_e^2}{2\pi m_s^4}\mu _{ea}^2\left( \frac{m_s^2}{4m_a^2}\right) , \end{aligned}$$

where $\mu _{ea}\equiv m_am_e/\left( m_a+m_e\right)$ is the reduced mass.

In Fig. 6, we show the 95 % expected sensitivity reached after 1 kg$\cdot$year exposure, corresponding to the cross section required to obtain 3.6 signal events [98] supposing a detector sensitivity to recoil energies between 1 meV and 1 eV [97]. One can see that even for quite low values of the coupling $g_e$, the prospect of discovery of self-interacting dark matter through this new proposal is quite promising.

## Conclusion

In this work, we have considered a pseudo-scalar dark matter candidate generated by the breaking of the global *U*(1) symmetry. In this framework, we have shown that one can compellingly combine the X-ray lines generated by annihilating warm dark matter to its self-interacting cross section. As a result, we have obtained the limits on the BSM scale $\Lambda \gtrsim 10^5-10^6~\mathrm {GeV}$ and on the dark matter mass $10~\mathrm {keV}\lesssim m_a\lesssim 10~\mathrm {MeV}$ depending on the fixed self-interacting cross section and the mass ratio $m_s/m_a$.

Moreover, we have done another combined analysis by fixing the annihilation cross section in order to reproduce the recent $3.5~\mathrm {keV}$ line claims. Surprisingly, a self-interacting cross section $\sigma /m$ of the order of 0.1–1 $\mathrm {cm^2/g}$ corresponding to recent claims from the observation of the cluster Abell 3827 induces naturally a keV line signal corresponding to the one which seems to have been observed in different clusters of galaxies like Perseus. Fitting both signals requires a BSM scale of the order of 100 TeV which could have some consequences for future accelerator searches. We have also discussed the non-detection of the X-ray lines from the Milky Way and stacked dwarf galaxies and found that they give a very strong constraint on the BSM scale $\Lambda$.

Such a light dark matter can be explored by the recent proposed direct detection technique through the coupling with electron. Even for the small coupling assumed, the detectability of the light dark matter candidate is promising due to the high sensitivity.

## Appendix

For completeness, here we present the main alternatives to the pseudo-scalar dark matter candidate, in the same framework.

### Majorana dark matter with scalar mediator

The interaction between a Majorana dark matter and a scalar mediator *s* is given by

17 $$\begin{aligned} \mathcal {L}=-\frac{g_\chi }{2} s\overline{\chi ^c}\chi +\frac{s}{\Lambda }F_{\mu \nu }F^{\mu \nu }, \end{aligned}$$

with the coupling $g_\chi$.

The self-interacting cross section of Majorana dark matter is computed from the Yukawa interaction. Although there are three contributions to the amplitude, coming from the *s*-, *t*-, and *u*-channels, the *s*-channel is velocity suppressed and the *t*- and *u*-channels give a dominant contribution. Thus the self-interacting cross section is given by

18 $$\begin{aligned} \sigma _{\chi \chi }=\frac{g_\chi ^4}{8\pi m_\chi ^2}\frac{m_\chi ^4}{m_s^4}, \end{aligned}$$

where $m_\chi$ is the dark matter mass.

For the annihilation process $\chi \chi \rightarrow \gamma \gamma$, only the *s*-channel contributes and the cross section is given by [36]

19 $$\begin{aligned} \sigma {v}_{\gamma \gamma }=\frac{g_\chi ^2}{\pi \Lambda ^2}\frac{m_\chi ^4v^2}{(4m_\chi ^2-m_s^2)^2}. \end{aligned}$$

As one can see from the formula, this cross section is suppressed by the dark matter relative velocity $v\sim 10^{-3}$. Thus it would be difficult to give a connection between the self-interacting dark matter and the X-ray monochromatic line unless an enhancement mechanism like Sommerfeld’s is taken into account.

### Majorana dark matter with pseudo-scalar mediator

For a Majorana dark matter $\chi$ interacting with a pseudo-scalar *a*, the Lagrangian is given by

20 $$\begin{aligned} \mathcal {L}=-\frac{\tilde{g}_\chi }{2} a\overline{\chi ^c}\gamma _5\chi +\frac{a}{\Lambda }F_{\mu \nu }\tilde{F}^{\mu \nu }, \end{aligned}$$

with the coupling $\tilde{g}_\chi$ where $\tilde{F}^{\mu \nu }\equiv \epsilon ^{\mu \nu \rho \sigma }F_{\rho \sigma }/2$ is the dual tensor of $F_{\mu \nu }$. There are *s*-, *t*- and *u*-channels contributing to the amplitude for the self-interacting cross section of dark matter. The amplitudes coming from the *t*- and *u*-channels are velocity suppressed and negligible. As a result, the dark matter self-interacting cross section is given by

21 $$\begin{aligned} \sigma _{\chi \chi }=\frac{\tilde{g}_\chi ^4}{8\pi m_\chi ^2}\frac{m_\chi ^4}{(4m_\chi ^2-m_a^2)^2}. \end{aligned}$$

The annihilation cross section for $\chi \chi \rightarrow \gamma \gamma$ mediated by the pseudo-scalar *a* is computed similarly to the scalar mediator case [36]:

22 $$\begin{aligned} \sigma {v}_{\gamma \gamma }= \frac{4\tilde{g}_\chi ^2}{\pi \Lambda ^2}\frac{m_\chi ^4}{(4m_\chi ^2-m_a^2)^2}. \end{aligned}$$

From Eqs. (21) and (22), one obtains

23 $$\begin{aligned} \sigma {v}_{\gamma \gamma }= & {} 8\sqrt{\frac{2}{\pi }}\frac{m_\chi ^{3/2}}{\Lambda ^2} \frac{m_\chi ^2}{m_a^2}\sqrt{\frac{\sigma _{\chi \chi }}{m_\chi }}\nonumber \\\simeq & {} 2.6\times 10^{-33} \left( \frac{10~\mathrm {TeV}}{\Lambda }\right) ^2 \left( \frac{E_\gamma }{3~\mathrm {keV}}\right) ^{3/2}\nonumber \\&\times \left( \frac{m_\chi /m_a}{0.1}\right) ^2 \sqrt{\frac{\sigma _{\chi \chi }/m_\chi }{1~\mathrm {cm^2/g}}}~\mathrm {cm^3/s}. \end{aligned}$$

where $m_\chi \ll m_a$ is assumed. One can see that $\Lambda$ is one order of magnitude smaller than the scalar dark matter case because of the additional suppression due to the ratio of the squared mass $m_\chi ^2/m_a^2$. However, this candidate is also a viable one, and it potentially explains the monochromatic signal and the self-interacting dark matter in a single framework.

## Footnotes

1 To keep the analysis as fair as possible, it is important to underline that there are still on-going debates on the possibility of explaining the X-ray line excess with thermal atomic transitions [61].

2 Interestingly, the authors in [71] addressed a similar issue in the case of exciting dark matter and long-range interaction. Our framework, being annihilating dark matter and contact interaction, our model, discussions, results, and prospects are completely different.

3 We neglected throughout our study the possible Higgs mixing as recent analysis on the invisible width of the Higgs impose stringent constraints on such mixings [72].

4 See [73] for a concrete example in the same framework where it has been shown that, in the meantime, a hierarchy $m_a \ll m_s$ is generated by the mechanism.

5 It is also interesting to note the possibility to obtain in the meantime the suitable relic abundance of dark matter without affecting $N_\mathrm {eff}$ if one adds a coupling to the neutrino sector as was shown in [36].

6 It is interesting to note that, in the meantime, the same authors derived recently before a stringent bound on the self-interacting scenario by the observation of 72 clusters collisions of $\sigma /m \lesssim 0.47 ~\mathrm {cm^2/g}$ [80].

7 Note that the *aas* coupling in the scalar potential could also affect the HB bound, since a pair of dark matter can be generated from the off-shell produced scalar *s*. However, this off-shell contribution is expected to be much smaller than the dominant Primakoff effect.

8 Another possibility would have been to look at the Centaurus [92] with $M_{Ce} = 6.3 \times 10^{13} M_{\odot }$ and a radius of $R_{Ce} = 0.17$ Mpc.
